# Supplementary material for: Integration of metagenome-assembled genomes with clinical isolates expands the genomic landscape of gut-associated Klebsiella pneumoniae
Source: Nat Commun. 2025 Nov 12;16:9959. doi: 10.1038/s41467-025-64950-6 (PMC12612154; doi:10.1038/s41467-025-64950-6)
Supplement: Supplementary file 1 — Supplementary Information [file 41467_2025_64950_MOESM1_ESM.pdf]

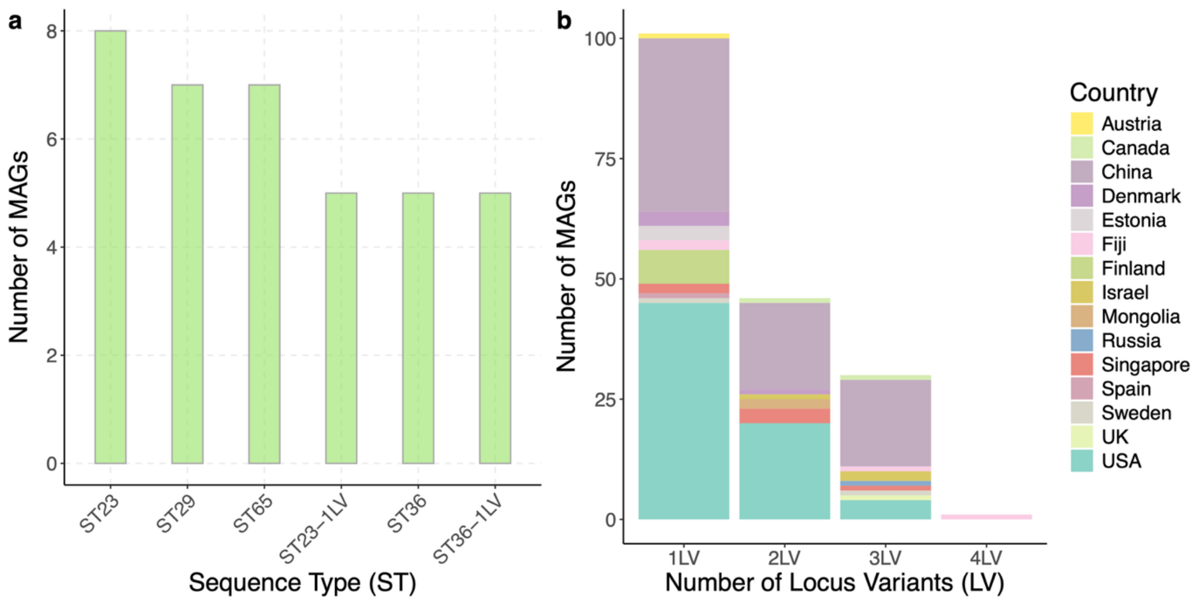

Supplementary Figure 1. Genotyping of MAGs with low strain heterogeneity. a, Most prevalent STs of the gut-derived *K. pneumoniae* metagenome-assembled genomes (MAGs) with an estimated strain heterogeneity <0.5%. **b**, Distribution of MAGs detected per country based on the number of locus variants (mutations) identified in the MLST genes in relation to a known ST profile. Only MAGs with strain heterogeneity <0.5% were considered.

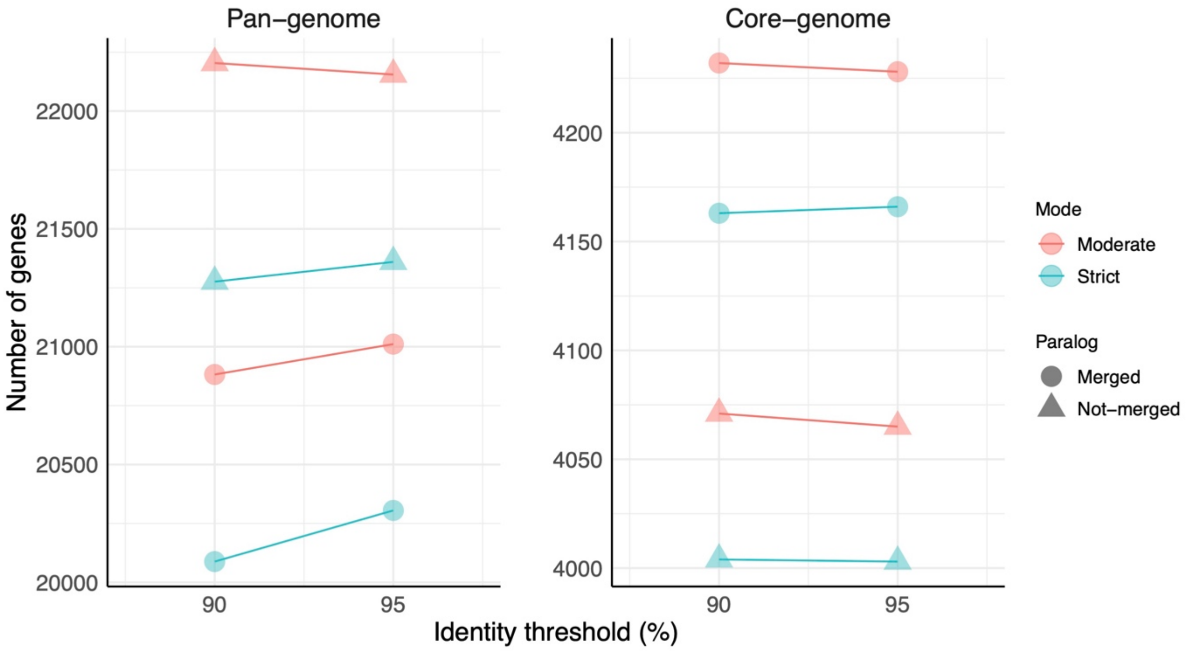

Supplementary Figure 2. Pan-genome size and number of core genes identified with Panaroo. Variation in pan-genome size and total number of core genes identified in *K. pneumoniae* according to different parameters used within Panaroo. The Y-axis represents the number of genes and the X-axis the two sequence identity thresholds (90% or 95%). The colour denotes the mode used (that is, strict or moderate) and the shapes the paralog handling method (merged or not merged). A core genome threshold was set at 90%.

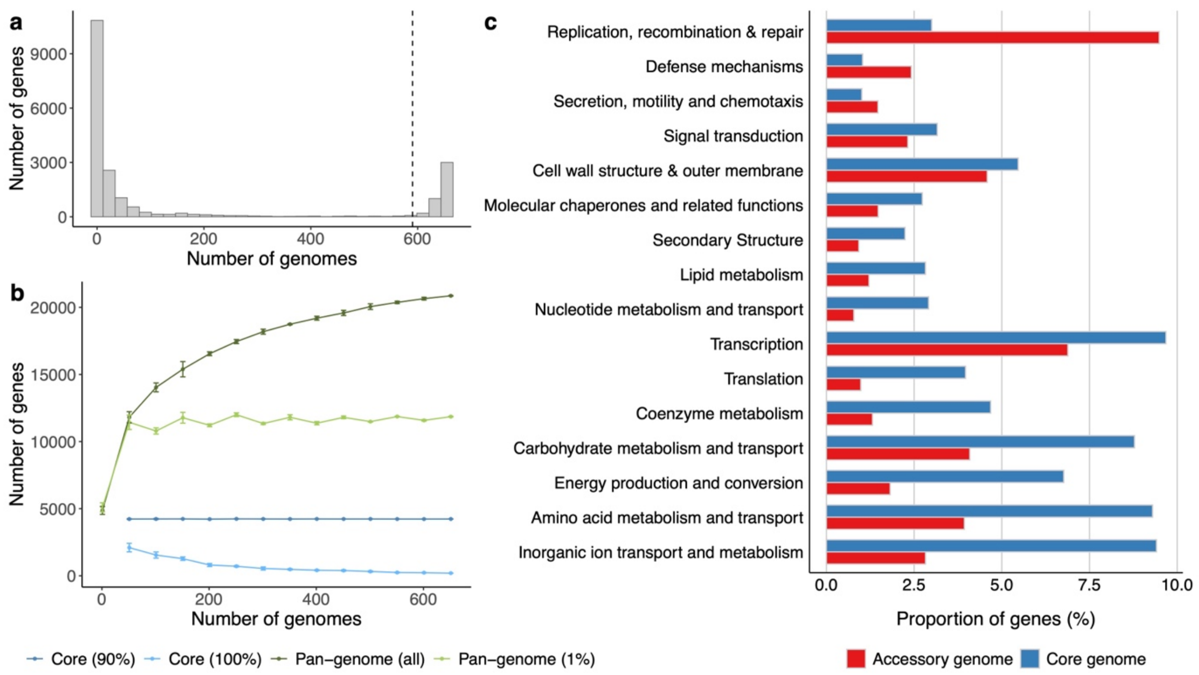

Supplementary Figure 3. Pan-genome patterns of *K. pneumoniae*. a, Distribution of the number of genes detected according to the number of *K. pneumoniae* genomes they were found in. A threshold of 90% (vertical line) was used to define core genes. **b,** Core- and pan-genome accumulation curves obtained according to different filtering thresholds. **c,** Functional categories differentially abundant between the accessory and core genome, tested with a two-sided Fisher's exact test (adjusted $P < 0.05$).

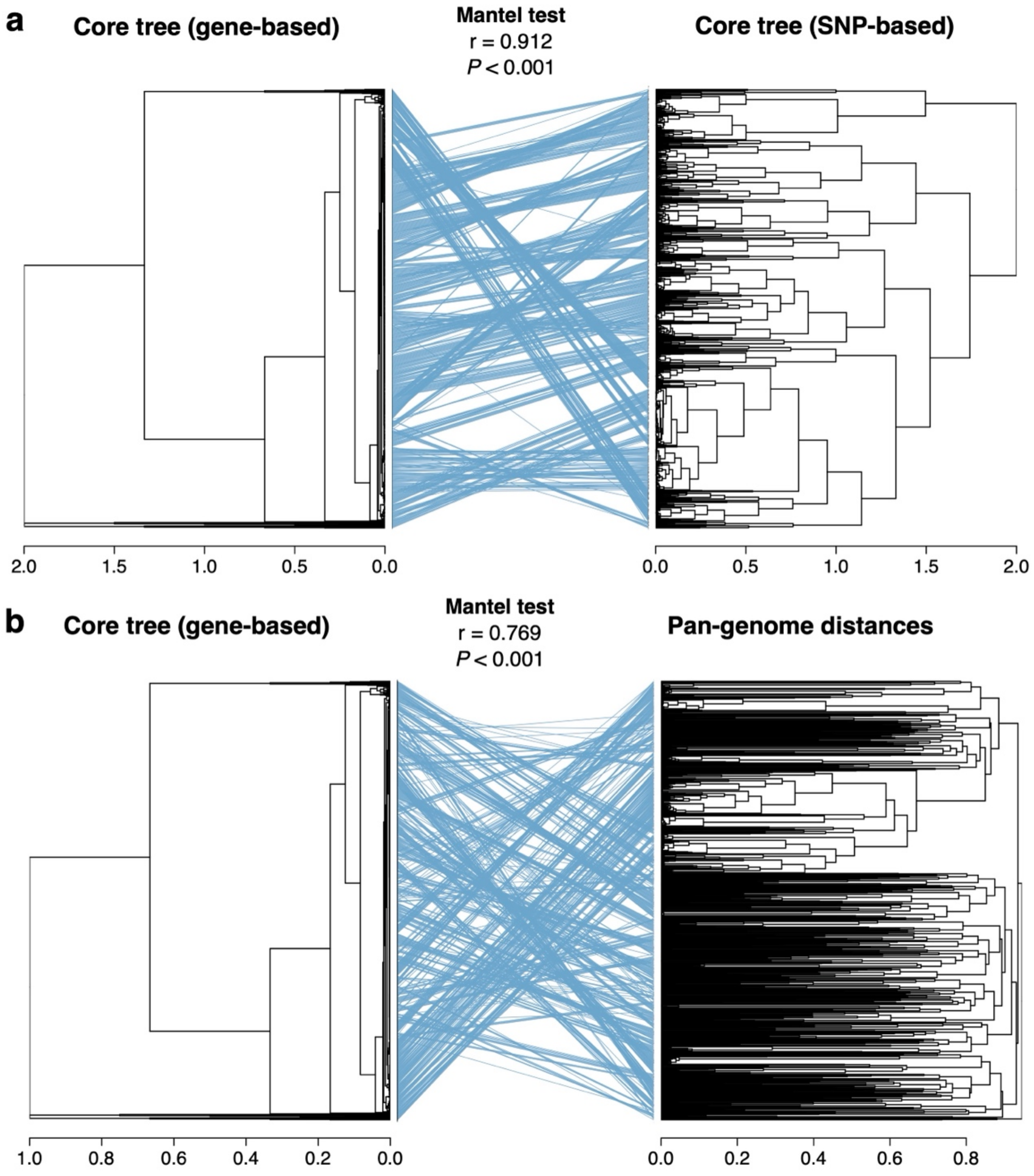

Supplementary Figure 4. Correlation between phylogenetic clustering methods. a, Tanglegram comparing the gene-based core phylogenetic tree of *K. pneumoniae* generated by Panaroo against the SNP-based core tree obtained with Snippy. **b**, Comparison of the gene-based core phylogenetic tree against the genome clustering obtained with Jaccard pan-genome distances (gene presence/absence patterns). A Mantel test was used to assess the correlation between each pair of trees.

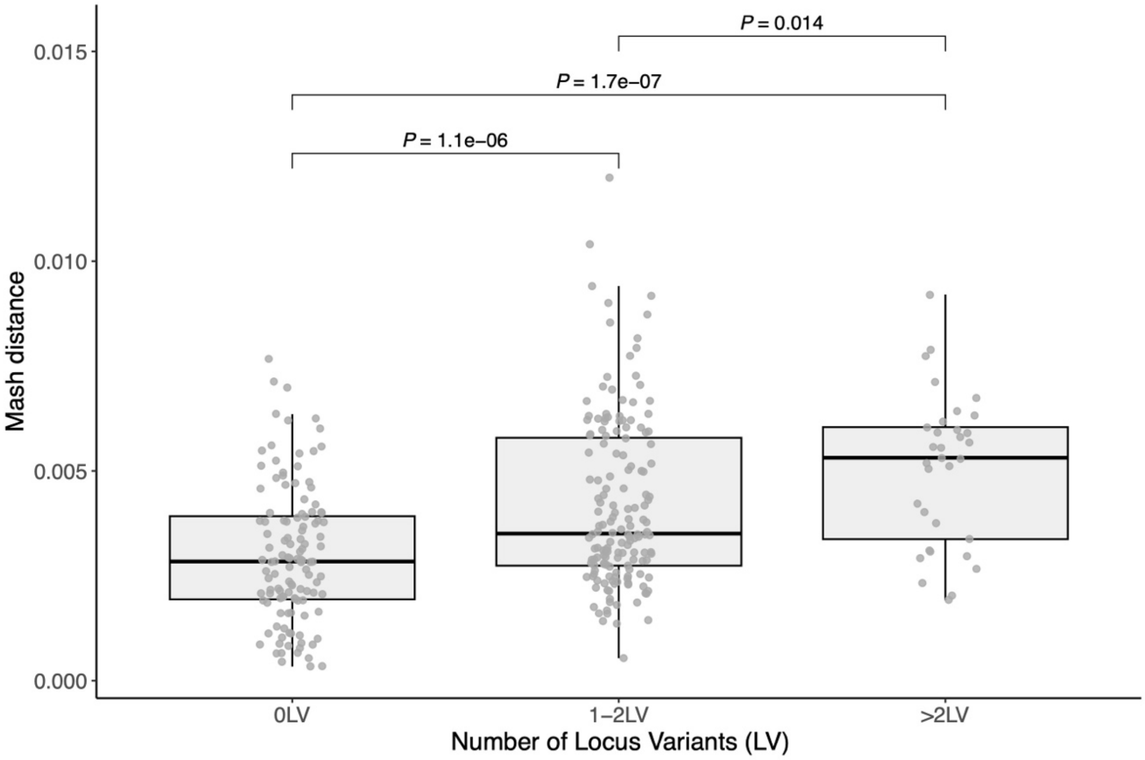

Supplementary Figure 5. Comparison of ST variation with genomic distance. Comparison of the distribution of Mash genomic distances estimated for metagenome-assembled genomes (MAGs) with no ST locus variants (0LV, n = 120), 1-2 LVs (n = 160) and >2LV (n = 33). Mash distances were inferred by comparing each MAG against their best-matching RefSeq genome. The centre line within the box represents the median score. Whiskers are shown extending to the furthest point within 1.5 times the IQR from the box. *P* values were derived from a two-sided Wilcoxon rank-sum test and corrected for multiple testing using the Bonferroni–Holm method.

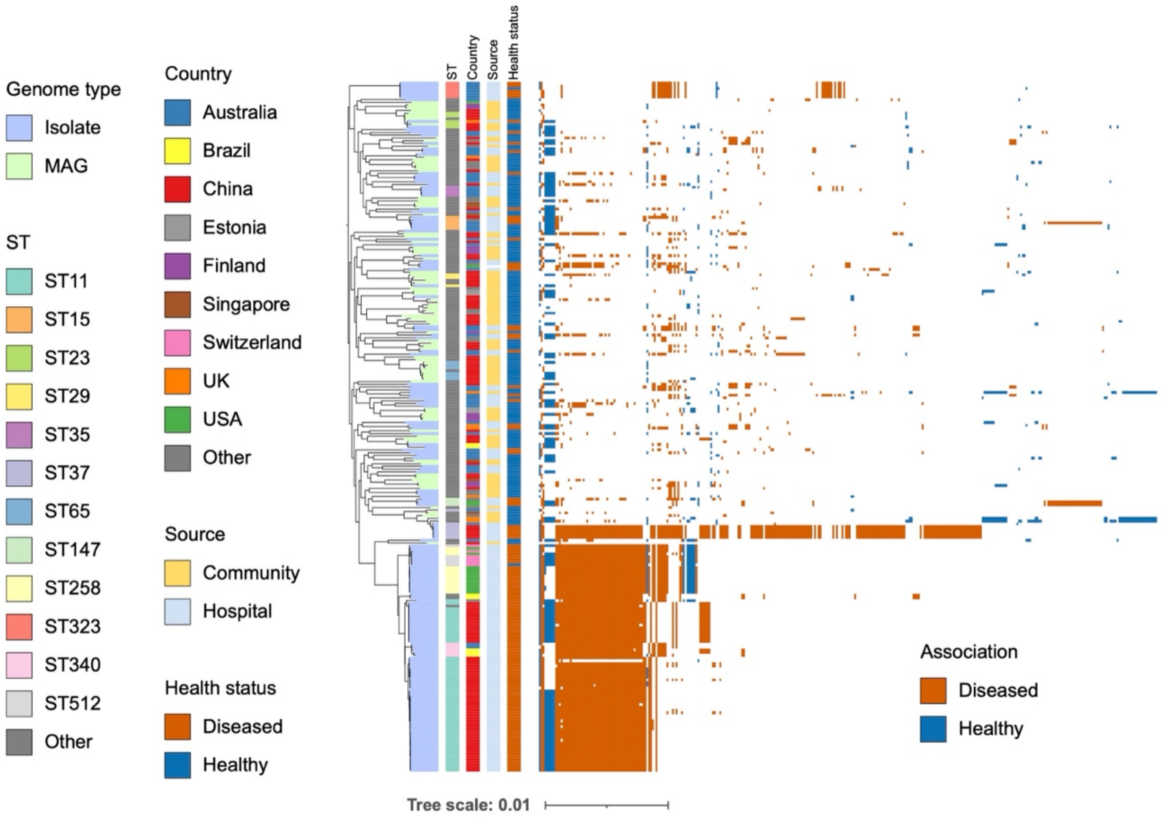

Supplementary Figure 6. Phylogenetic tree and candidate genes linked to carriage or infection. Core-genome phylogenetic tree of *K. pneumoniae* genomes from carriage and infection. The first four annotation blocks denote various metadata properties of the genomes, while the remaining layers to the right depict the distribution of all the significant genes associated with either carriage or infection.

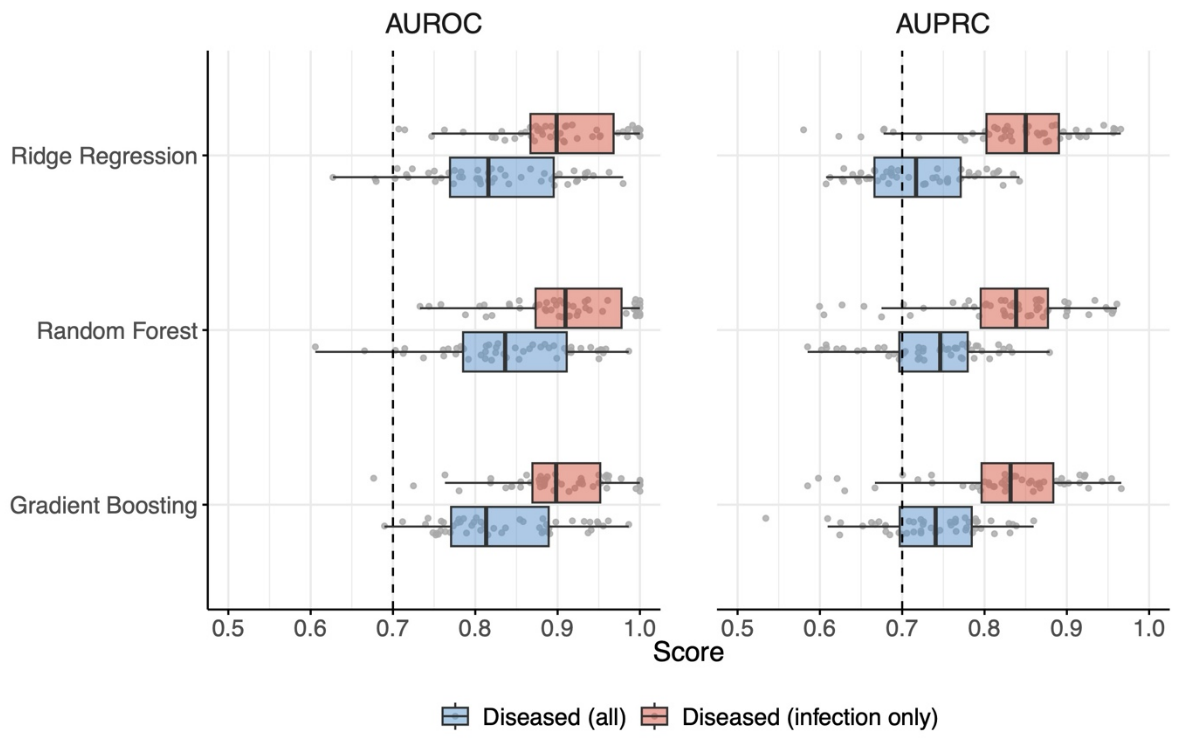

Supplementary Figure 7. Classification of carriage and disease genomes using supervised machine learning models. Performance of machine learning (ML) models distinguishing carriage- from disease-associated *K. pneumoniae*, considering either all disease genomes (blue) or just those from infection (red). Three supervised ML models were tested (*n* = 50 seeds per model): ridge regression, random forest and gradient boosting. Each box in the plot represents the interquartile range (IQR) of the Area Under the Receiver Operating Characteristic Curve (AUROC, left) or the Area Under the Precision-Recall Curve (AUPRC, right). The centre line within the box represents the median score. Whiskers are shown extending to the furthest point within 1.5 times the IQR from the box.

Supplementary Table 1. PERMANOVA results assessing the relationship between metadata variables and genome clustering methods.

Analysis	Variable	R^2	P-value	Df	N
Core tree (gene-based)	Continent	3.53511137	0.001	5	630
Core tree (gene-based)	Country	9.8696037	0.001	28	630
Core tree (gene-based)	Genome type	5.61048292	0.001	1	656
Core tree (gene-based)	Source	1.94117772	0.001	1	629
Core tree (gene-based)	Health status (generic)	5.34988967	0.001	2	511
Core tree (gene-based)	Health status (specific)	11.2672237	0.001	19	511
Core tree (SNP-based)	Continent	4.13258652	0.001	5	630
Core tree (SNP-based)	Country	11.2628136	0.001	28	630
Core tree (SNP-based)	Genome type	6.30029128	0.001	1	656
Core tree (SNP-based)	Source	2.34908964	0.001	1	629
Core tree (SNP-based)	Health status (generic)	6.53350482	0.001	2	511
Core tree (SNP-based)	Health status (specific)	12.8830911	0.001	19	511
Pan-genome	Continent	5.54959407	0.001	5	630
Pan-genome	Country	12.8484946	0.001	28	630
Pan-genome	Genome type	7.61411467	0.001	1	656
Pan-genome	Source	3.09751934	0.001	1	629
Pan-genome	Health status (generic)	6.31992014	0.001	2	511
Pan-genome	Health status (specific)	15.5062612	0.001	19	511
