# Supplementary material for: Integration of metagenome-assembled genomes with clinical isolates expands the genomic landscape of gut-associated Klebsiella pneumoniae
Source: Nat Commun. 2025 Nov 12;16:9959. doi: 10.1038/s41467-025-64950-6 (PMC12612154; doi:10.1038/s41467-025-64950-6)
Supplement: Supplementary file 2 — Description of Additional Supplementary Files [file 41467_2025_64950_MOESM2_ESM.pdf]

Description of Additional Supplementary Files

File Name: Supplementary Data 1.

Description: Metadata and genome properties of the *Klebsiella pneumoniae* genomes analysed in this study.

File Name: Supplementary Data 2.

Description: Annotation of genes identified exclusively among the *Klebsiella pneumoniae* metagenome-assembled genomes (MAGs) analysed in this study.

File Name: Supplementary Data 3.

Description: Candidate genes derived from the genome-wide association study comparing *Klebsiella pneumoniae* lineages between carriage and disease. AF = Allele Frequency.
