# Supplementary material for: Integration of metagenome-assembled genomes with clinical isolates expands the genomic landscape of gut-associated Klebsiella pneumoniae
Source: Nat Commun. 2025 Nov 12;16:9959. doi: 10.1038/s41467-025-64950-6 (PMC12612154; doi:10.1038/s41467-025-64950-6)
Supplement: Supplementary file 6 — Reporting Summary [file 41467_2025_64950_MOESM6_ESM.pdf]

nature portfolio

Corresponding author(s): Alexandre Almeida

Last updated by author(s): Sep 26, 2025

Reporting Summary

Nature Portfolio wishes to improve the reproducibility of the work that we publish. This form provides structure for consistency and transparency in reporting. For further information on Nature Portfolio policies, see our Editorial Policies and the Editorial Policy Checklist.

Statistics

For all statistical analyses, confirm that the following items are present in the figure legend, table legend, main text, or Methods section.

n/a	Confirmed	
☐	☒	The exact sample size (*n*) for each experimental group/condition, given as a discrete number and unit of measurement
☒	☐	A statement on whether measurements were taken from distinct samples or whether the same sample was measured repeatedly
☐	☒	The statistical test(s) used AND whether they are one- or two-sided<br>*Only common tests should be described solely by name; describe more complex techniques in the Methods section.*
☐	☒	A description of all covariates tested
☐	☒	A description of any assumptions or corrections, such as tests of normality and adjustment for multiple comparisons
☐	☒	A full description of the statistical parameters including central tendency (e.g. means) or other basic estimates (e.g. regression coefficient) AND variation (e.g. standard deviation) or associated estimates of uncertainty (e.g. confidence intervals)
☐	☒	For null hypothesis testing, the test statistic (e.g. *F*, *t*, *r*) with confidence intervals, effect sizes, degrees of freedom and *P* value noted<br>*Give P values as exact values whenever suitable.*
☒	☐	For Bayesian analysis, information on the choice of priors and Markov chain Monte Carlo settings
☒	☐	For hierarchical and complex designs, identification of the appropriate level for tests and full reporting of outcomes
☐	☒	Estimates of effect sizes (e.g. Cohen's *d*, Pearson's *r*), indicating how they were calculated

Our web collection on statistics for biologists contains articles on many of the points above.

Software and code

Policy information about availability of computer code

Data collection	No software was used for data collection
Data analysis	CheckM v.1.0.11; GUNC v.1.0.3; Kleborate v.2.4.1; Panaroo v.1.3.3; eggNOG-mapper v.2.1.3; Snippy v.4.6.0; Gubbins v.3.3.1; FastTree v.2.1.11; R packages (dendextend, chronos, ape, phytools, vegan, mikropml); Pyseer v.1.3.11; AMRFinderPlus v.3.11.26; DeepARG v.1.0.4; DeepVF (release 2019-08-30). KpMAGs GitHub: https://github.com/microfundiv-lab/KpMAGs

For manuscripts utilizing custom algorithms or software that are central to the research but not yet described in published literature, software must be made available to editors and reviewers. We strongly encourage code deposition in a community repository (e.g. GitHub). See the Nature Portfolio guidelines for submitting code & software for further information.

Data

Policy information about availability of data

All manuscripts must include a data availability statement. This statement should provide the following information, where applicable:

- Accession codes, unique identifiers, or web links for publicly available datasets
- A description of any restrictions on data availability
- For clinical datasets or third party data, please ensure that the statement adheres to our policy

All genomes used are publicly available in the Unified Human Gastrointestinal Genome catalogue (https://ftp.ebi.ac.uk/pub/databases/metagenomics/mgnify_genomes/human-gut/v1.0/) and the European Nucleotide Archive (see Supplementary Table 1 for list of accession codes and genome identifiers). Pan-genome data files (gene presence/absence matrix and FASTA file) are available in: https://doi.org/10.6084/m9.figshare.27961089.v2.

Research involving human participants, their data, or biological material

Policy information about studies with human participants or human data. See also policy information about sex, gender (identity/presentation), and sexual orientation and race, ethnicity and racism.

Reporting on sex and gender	N/A
Reporting on race, ethnicity, or other socially relevant groupings	N/A
Population characteristics	N/A
Recruitment	N/A
Ethics oversight	N/A

Note that full information on the approval of the study protocol must also be provided in the manuscript.

Field-specific reporting

Please select the one below that is the best fit for your research. If you are not sure, read the appropriate sections before making your selection.

[x] Life sciences   [ ] Behavioural & social sciences   [ ] Ecological, evolutionary & environmental sciences

For a reference copy of the document with all sections, see nature.com/documents/nr-reporting-summary-flat.pdf

Life sciences study design

All studies must disclose on these points even when the disclosure is negative.

Sample size	We extracted all high-quality Klebsiella pneumoniae genomes derived from faecal samples available in the Unified Human Gastrointestinal Genome (UHGG) v1.0 database (n=656). To augment our dataset, we further compared this genome set against all Klebsiella genomes available in RefSeq (n=20,792). Statistical analyses revealed these numbers were sufficient to identify genomic differences between carriage- and disease-associated genomes.
Data exclusions	We applied a filter of >90% completeness and <5% contamination based on the genome statistics obtained with CheckM v1.0.11. Thereafter, we used GUNC v1.0.3 to further exclude genomes with both a 'clade_separation_score' >0.45 and 'contamination_portion' >0.05. Lastly, all genomes underwent taxonomic verification for K . pneumoniae using Kleborate v2.4.1 using default parameters.
Replication	Machine learning models were trained on 80% of the data and tested on the remaining 20%. To ensure reproducibility, each model (ridge regression, random forest or gradient boosting) was repeated with 50 independent seeds. Several of our analyses (ST distribution, genome signatures of health and disease) were repeated using genomes collected from countries where genomes from both sample groups were obtained.
Randomization	Genomes were classified into carriage or disease based on the health status reported for the individual sampled. Population structure, country of origin and genome type (MAG or isolate) were controlled for in both the genome-wide association study analysis and the machine learning modelling.
Blinding	Blinding was not relevant to this study, as genomes were grouped and categorized into carriage or disease based on available metadata.

Reporting for specific materials, systems and methods

We require information from authors about some types of materials, experimental systems and methods used in many studies. Here, indicate whether each material, system or method listed is relevant to your study. If you are not sure if a list item applies to your research, read the appropriate section before selecting a response.

Materials & experimental systems

n/a	Involved in the study	
[x]	[ ]	Antibodies
[x]	[ ]	Eukaryotic cell lines
[x]	[ ]	Palaeontology and archaeology
[x]	[ ]	Animals and other organisms
[x]	[ ]	Clinical data
[x]	[ ]	Dual use research of concern
[x]	[ ]	Plants

Methods

n/a	Involved in the study	
[x]	[ ]	ChIP-seq
[x]	[ ]	Flow cytometry
[x]	[ ]	MRI-based neuroimaging

Plants

Seed stocks	N/A
Novel plant genotypes	N/A
Authentication	N/A
